# A supplier selection model in pharmaceutical supply chain using PCA, Z-TOPSIS and MILP: A case study

**DOI:** 10.1371/journal.pone.0201604

**Published:** 2018-08-15

**Authors:** Athena Forghani, Seyed Jafar Sadjadi, Babak Farhang Moghadam

**Affiliations:** 1 Department of Industrial Engineering, Science and Research Branch, Islamic Azad University, Tehran, Iran; 2 Department of Industrial Engineering, Iran University of Science and Technology, Tehran, Iran; 3 Institute for Management and Planning Studies, Tehran, Iran; Southwest University, CHINA

## Abstract

Supplier selection is one of the critical processes in supplier chain management which is associated with the flow of goods and services from the supplier of raw material to the final consumer. The purpose of this paper is to present a novel approach and improves the supplier selection in a multi-item/multi-supplier environment, and provide the importance and the reliability of the criteria by handling vagueness and imperfection of information in decision making process. First, principal component analysis (PCA) method is used to reduce the number of supplier selection criteria in pharmaceutical companies. Next, using the most important criteria resulted from the PCA method, the importance and the reliability of the selected criteria are assessed by a group of decision-maker (DM). Then, the importance value of each supplier with respect to each product is obtained via the Technique for Order Performance by Similarity to Ideal Solution (TOPSIS) based on the concept of Z-numbers called Z-TOPSIS. Finally, these values are used as inputs in a mixed integer linear programming (MILP) to determine the suppliers and the amount of the products provided from the related suppliers. To validate the proposed methodology, an application is performed in a pharmaceutical company. The results show that the proposed method could provide promising results in decision making process more appropriately.

## Introduction

Supplier selection is one of the most important activities for most enterprises and has a substantial impact on the efficiency and effectiveness of the entire supply chain [1,2]. It is likely that the manufacturer allocates more than 60% of its total sales on purchased services and materials [3]. Furthermore, material cost is up to 70% of the finished product expenses [4]. Therefore, selecting the appropriate suppliers can result in reduced purchasing cost, decreased supplying risk and improved product quality [5].

When it comes to select a suitable supplier, various criteria need to be contemplated. However, it would not be appropriate to recommend and sometimes even it would not be possible to take into account all the criteria upon final decision making due to the diversity of strategies among the industries concerning the supply chain regarding the product's characteristics.

Typically in dealing with the supplier selection discussion, two kinds of problems are propounded: First, the supplier is the one (natural or legal entity) who satisfies all the purchasers' requirements (single sourcing); in this type of supplier selection, the management should make the only decision to determine the best supplier. Second, there is no single supplier to meet all the purchasers' needs (multiple sourcing). Many companies encounter some disruption or inadequate supply capacity on the part of the supplier. Adopting the second model, which is the ‘multiple sourcing’ the purchasing company meanwhile using the business process, can resolve the unpredicted delay of supply by one of the numerous other suppliers.

It would not be convenient for the DM to choose suitable suppliers who can fulfill all the firms' demands based on different criteria. Another point to consider is that as a multiple criteria decision-making (MCDM) problem, the supplier selection would lie under the effect of many qualitative and quantitative contradictory factors. In order to maintain an equilibrium among such conflicting criteria, many studies have proposed various models, encompassing single to hybrid approaches. Adopting the single approach, the studies often consider locating a solution for the supplier selection problem through specifying the optimal quantities that are usually under the effect of a number of constraints. As an example, Zhang and Zhang [6] in their study for minimizing the total cost including the product and fixed costs with stochastic demand, developed a mixed-integer programming (MIP) model based on the assumption that all the existing suppliers could meet the qualitative criteria level. In another study on the supplier selection problem, Du *et al*. [7] considered the life-cycle cost through developing a bi-objective model that could account for the operational cost together with the purchasing cost since decreasing only the purchasing cost may result in more frequent failure of equipment, which results in increase of the maintenance costs. For model solving, they introduced a Pareto genetic algorithm hybridization, multi-intersection and similarity crossover strategy. In order to minimize the total cost of the product and maximize the quality of the products as well as the reliability of delivery, Karpak *et al*. [8] used goal programming (GP) in hydraulic pump division of a US based manufacturer. Karpak *et al*. [9] employed visual interactive GP in finding solution for single- and multiple-product supplier selection problems. In order to select the suppliers and allocating the required quantities based on the total cost of product, total quality and delivery reliability, Karpak *et al*. [10] designed a GP model subject to demand and capacity constraints. Fuzzy mixed-integer GP was developed by Kumar *et al*. [11] for solving the supplier selection problem of fuzzy nature. An MIP model for stochastic supplier selection was introduced by Amorim *et al*. [12] in the food industry. A Monte Carlo simulation was applied for fuzzy GP by Moghaddam [13] for the purpose of solving the supplier selection problem.

Amin *et al*. [14] was the first to consider the strategic perspectives in applying hybrid models by devising a two-stage integrated quantified SWOT analysis technique with fuzzy linear programming (fuzzy LP) to resolve the supplier selection problem. A number of studies have attempted to apply historical data for supplier selection problem; Faez *et al*. [15] for example introduced an integrated case-based reasoning with MIP for selecting the supplier and the required quantities of goods to order. An integrated approach of AHP, enhanced by rough set theory and multi-objectives mixed-integer linear programming (MILP), was introduced by Xia and Wu [16] for a multi-product supplier selection and order allocation problem, in which the suppliers will offer price discounts on sum of the trade volumes.

Demirtas and Üstün [17], based on the analytical network process (ANP) and the multi-objective mixed-integer programming (MOMIP), developed a two-stage supplier selection and order allocation model to minimize the purchasing value, the budget and defect rates. Using the Tchebycheff procedure the model was solved by the ϵ-constraint method and the reservation level. In order to deal with the supplier selection problem, Wu *et al*. [18] used the Delphi method, ANP, and the MOMIP model for supplier selection. In this model the criteria are first generated by experts using the Delphi method. Then, the obtained criteria are served as input for the ANP method and in the end, the MOMIP model is utilized for selecting the best suppliers and the relevant quantities.

Additionally, in a study undertaken by Lee *et al*. [19] the fuzzy AHP and fuzzy multiple goal planning were used for selecting the suppliers of a company producing thin-film-transistor liquid-crystal display products. A two-stage model was developed by Liao and Kao [20] by applying fuzzy TOPSIS and multi-choice GP for selection the appropriate suppliers and allocating the orders. Also fuzzy TOPSIS and multi-choice GP were used by Rouyendegh and Saputro [21] in a fertilizer and chemical producing company. In another study conducted by Kilic [22], the fuzzy TOPSIS was employed together with MILP for selecting the best supplier in a multi-item/multi-supplier problem. Perçin [23] used integrated AHP–GP for supplier selection problem. SWOT analysis was used in a study carried out by Ghorbani *et al*. [24] for evaluating the suppliers; they also used integer linear programming (ILP) model for selecting and determining the quantities. The group decision making with different voting power and linear programming (LP) were used by Sodenkamp *et al*. [25] for supplier selection problem. Simić [26] has reviewed the 50 years of fuzzy set theory and models for supplier selection.

This paper intends to adopt a qualitative method by using PCA, Z-TOPSIS algorithm [27] with triangular fuzzy number and a mixed integer linear programming for supplier selection. In order to show its applicability, the proposed methodology is implemented using a case study involving a pharmaceutical company.

The rest of the paper is organized as follows: Sections 2 and 3 present the supplier criteria in pharmaceutical companies and a questionnaire to gather information about the importance of the criteria; Section 4 reviews the methodology of PCA and its application in reducing the number of the criteria; Section 5 briefly reviews Z-TOPSIS method; Section 6 presents a new model; Section 7 gives a numerical example to show applicability of the proposed model; Section 8 presents sensitivity analysis of the results; and Section 9 concludes the paper.

## Supplier criteria

In pharmaceutical companies, the main criteria are grouped under six categories, which are cost, quality, services, delivery, supplier profile and overall personnel capabilities. These primary criteria are decomposed into various sub-criteria as represented in Table 1:

**Table 1:** Supplier selection criteria in pharmaceutical companies.

Main Criteria	Sub-criteria	
Cost	*c*_1_	product price
	*c*_2_	Payment terms
	*c*_3_	Delivery cost
Quality	*c*_4_	Product quality
	*c*_5_	The number of the defective items
	*c*_6_	Packaging and labeling
	*c*_7_	ISO 9001 (quality management system certification)
	*c*_8_	Research, development and innovation
Services	c_9_	Customer relationship management (CRM)
	c_10_	After sales service/warranty
Delivery	c_11_	Geographical location
	c_12_	On time delivery
Supplier profile	c_13_	Financial status
	c_14_	Management and organization
	c_15_	Technical ability
	c_16_	Facilities
	c_17_	Capacity
	*c*_18_	Past record documentation
	*c*_19_	Certificate of GMP (Good Manufacturing Practice)
	*c*_20_	ISO 14001 (environmental management system certification)
	*c*_21_	OHSAS 18001 (occupational health and safety management system certification)
	*c*_22_	Risk assessment system
Overall personnel capabilities	*c*_23_	labor overall skills
	*c*_24_	labor experience

In the next section, a questionnaire is used to collect information about the importance of these 24 criteria. Then, the PCA method is applied to reduce the number of criteria to ease the methodology.

## Methods

To rate the supplier selection criteria (*c*_*1*_,*c*_*2*_,…,*c*_*24*_) from 0 (the least important) to 10 (the most important), we asked the business managers of 34 pharmaceutical companies: Hakim(*x*_*1*_), Aboureyhan(*x*_*2*_), Behvazan(*x*_*3*_), Akbarieh(*x*_*4*_), Arya(*x*_*5*_), Raha(*x*_*6*_), Aryo Gen(*x*_*7*_), Bakhtar Bioshimi(*x*_*8*_), Cosar(*x*_*9*_), Behsa(*x*_*10*_), Caspian Tamin(*x*_*11*_), Tehran Chemi(*x*_*12*_), Cobel Darou(*x*_*13*_), Doctor Abidi(*x*_*14*_), Exir(*x*_*15*_), Farabi(*x*_*16*_), Iran Najou(*x*_*17*_), Jaber Ebne Hayan(*x*_*18*_), Kish Medipharm(*x*_*19*_), Loghman(*x*_*20*_), Alborz Darou(*x*_*21*_), Osveh(*x*_*22*_), Pars Darou(*x*_*23*_), Ramofarmin(*x*_*24*_), Chemi Darou(*x*_*25*_), Razi(*x*_*26*_), Shahid Ghazy(*x*_*27*_), Sina Darou(*x*_*28*_), Behestan Darou(*x*_*29*_), Rooz Darou(*x*_*30*_), Sobhan Darou(*x*_*31*_), Zahraavi(*x*_*32*_), Iran Darou(*x*_*33*_), Shafa(*x*_*34*_).

Note that the research was accomplished during the year of 2017. The questionnaire (S1 File) was sent to the companies through e-mail address, and completion of the questionnaire was taken as consent. Table 2 presents the results of the completed questionnaires.

**Table 2:** Questionnaire.

	*x*_1_	*x*_2_	*x*_3_	*x*_4_	*x*_5_	*x*_6_	*x*_7_	*x*_8_	*x*_9_	*x*_10_	*x*_11_	*x*_12_	*x*_13_	*x*_14_	*x*_15_	*x*_16_	*x*_17_	*x*_18_	*x*_19_	*x*_20_	*x*_21_	*x*_22_	*x*_23_	*x*_24_	*x*_25_	*x*_26_	*x*_27_	*x*_28_	*x*_29_	*x*_30_	*x*_31_	*x*_32_	*x*_33_	*x*_34_
***c***_**1**_	10	9	10	9	8	10	10	10	10	9	10	10	10	10	9	10	9	10	9	10	10	10	9	10	7	10	9	10	10	9	9	10	10	9
***c***_**2**_	5	9	6	5	6	6	6	10	7	9	10	8	8	3	5	8	7	8	6	7	4	10	6	6	6	5	5	7	7	6	6	7	7	8
***c***_**3**_	6	8	6	4	6	7	6	10	7	10	10	6	8	6	4	4	6	9	4	7	5	10	7	7	7	6	6	7	7	3	5	7	7	7
***c***_**4**_	10	9	10	10	9	10	10	10	10	10	10	10	10	10	10	10	10	10	10	10	10	10	10	10	10	10	10	10	10	10	10	10	10	10
***c***_**5**_	3	6	6	2	6	3	5	8	5	7	10	4	7	3	2	7	10	7	5	6	4	10	6	9	6	3	6	6	7	6	6	6	10	9
***c***_**6**_	4	7	6	1	6	5	5	6	6	7	10	3	7	6	1	4	10	7	4	6	2	9	9	10	7	4	8	6	7	3	3	6	10	9
***c***_**7**_	4	8	6	4	6	2	5	8	4	6	8	3	7	6	4	3	10	7	5	6	5	10	8	8	7	4	10	6	7	4	4	6	7	9
***c***_**8**_	3	4	5	3	5	4	4	8	4	10	10	6	6	1	2	1	8	9	3	5	4	10	6	6	5	3	7	5	6	4	1	5	8	8
***c***_**9**_	9	10	8	10	7	9	8	9	10	10	10	7	8	9	10	8	10	9	10	10	8	10	9	10	8	9	10	10	10	9	8	10	10	8
***c***_**10**_	7	5	8	9	7	6	7	9	6	8	10	5	8	6	9	5	10	8	4	7	7	10	9	10	7	5	8	7	8	4	6	7	10	8
***c***_**11**_	3	8	2	0	4	5	3	10	2	7	6	0	4	0	1	0	3	7	1	2	2	7	8	5	3	1	6	3	4	2	1	3	6	8
***c***_**12**_	4	8	6	4	6	1	5	10	4	10	10	6	7	2	3	1	9	8	7	6	3	10	9	9	7	2	5	6	7	7	1	6	10	8
***c***_**13**_	6	10	6	7	6	6	6	8	7	7	6	6	8	4	7	4	8	6	6	7	5	10	7	8	7	3	8	7	7	5	5	7	10	8
***c***_**14**_	5	8	6	5	6	6	5	8	6	7	6	5	7	2	5	7	6	7	6	7	3	10	6	7	7	8	10	6	7	6	7	6	9	8
***c***_**15**_	7	7	7	6	8	4	7	7	7	8	7	8	8	6	8	7	10	7	8	8	6	10	8	10	7	7	10	8	8	6	8	8	10	10
***c***_**16**_	4	7	6	4	6	4	5	8	4	8	8	4	7	2	6	4	7	9	5	6	4	10	7	7	6	5	10	6	7	5	4	6	10	9
***c***_**17**_	2	8	6	5	6	4	5	8	4	8	8	4	7	5	6	5	9	9	5	6	2	10	7	7	6	5	9	6	7	4	4	6	10	9
***c***_**18**_	10	10	9	10	9	8	9	10	9	10	7	9	10	8	9	10	9	10	10	10	8	10	9	9	7	8	10	9	6	8	8	9	9	7
***c***_**19**_	10	10	10	10	10	10	10	10	10	10	10	10	10	10	10	10	10	10	10	10	10	10	10	10	10	10	10	10	10	10	10	10	10	10
***c***_**20**_	5	6	7	4	6	5	6	8	6	7	6	5	8	5	4	5	10	7	6	5	6	10	8	8	6	6	10	7	7	7	5	7	7	7
***c***_**21**_	3	6	4	3	4	2	4	6	2	6	4	3	6	3	3	4	9	7	3	3	3	8	8	8	4	3	10	5	5	3	4	5	6	7
***c***_**22**_	3	6	5	1	4	1	3	3	0	3	4	2	5	3	1	4	9	7	2	3	1	10	7	7	4	2	8	4	5	2	5	4	7	9
***c***_**23**_	6	9	6	6	6	7	7	5	5	5	6	4	8	5	7	5	10	8	7	7	5	10	6	6	6	6	8	7	7	7	5	7	10	8
***c***_**24**_	6	8	5	6	5	4	5	5	6	5	5	6	8	7	6	5	10	7	6	7	5	10	7	7	6	7	7	7	7	6	5	7	10	9

Next section reviews the methodology of PCA and its application in reducing the number of the supplier selection criteria.

### Principal component analysis (PCA)

Pearson [28] was the first to introduce the PCA for the dimensionality reduction purpose. With respect to PCA, two main ideas can be enumerated; first, it provides an efficient data analysis tool for identifying and expressing data patterns and determining data similarities as well as differences. Second, in terms of its ability for data compression through minimizing the data set dimensionality which is constituted from a numerous interrelated variables with almost zero information loss [29]. Data compression can be performed through original data transformation into a new collection of variables and in fact into new set of principal components (*ζ*_*s*_) virtually uncorrelated to each other. Depending on the level of significance, the *ζ*_*s*_ will be represented in declining order, so that only the first few number of important ones are retained. Using the PCA, the data dimensionality is decreased and the multicollinearity can be removed [30].

### Methodology

Let *x* = (*x*_*1*_,*x*_*2*_,…,*x*_*j*_,…,*x*_*p*_); and *n* as the number of observations allocated for each variable, hence the data matrix *x* would be:

$$x=(x_{1},x_{2},\ldots ,x_{j},\ldots ,x_{p})=\left[\begin{array}{cc} \begin{array}{ccc} X_{11} & X_{12} & \cdots \\ X_{21} & X_{22} & \cdots \\ \vdots & \vdots & \ddots \end{array} & \begin{array}{ccc} X_{1j} & \cdots & X_{1p}\\ X_{2j} & \cdots & X_{2p}\\ \vdots & \ddots & \vdots \end{array}\\ \begin{array}{ccc} X_{i1} & X_{i2} & \cdots \\ \vdots & \vdots & \ddots \\ X_{n1} & X_{n2} & \cdots \end{array} & \begin{array}{ccc} X_{ij} & \cdots & X_{ip}\\ \vdots & \ddots & \vdots \\ X_{n3} & \cdots & X_{np} \end{array} \end{array}\right]$$ (1)

The PCA tries to find a new set of variables *ζ*_*s*_, where *ζ*_*s*_ = (*ζ*_*1s*_,*ζ*_*2s*_,…,*ζ*_*ns*_) is the linear functions of *x*. It can be said that from *ζ*_*1*_ to *ζ*_*p*_, in descending order of importance, they are uncorrelated:

$$\left[\begin{array}{c} \zeta_{1}\\ \begin{array}{c} \zeta_{2}\\ \begin{array}{c} \vdots \\ \zeta_{i} \end{array}\\ \vdots \end{array}\\ \zeta_{p} \end{array}\right]=\left[\begin{array}{cc} \begin{array}{ccc} \alpha_{11} & \alpha_{12} & \cdots \\ \alpha_{21} & \alpha_{22} & \cdots \\ \vdots & \vdots & \ddots \end{array} & \begin{array}{ccc} \alpha_{1j} & \cdots & \alpha_{1p}\\ \alpha_{2j} & \cdots & \alpha_{2p}\\ \vdots & \ddots & \vdots \end{array}\\ \begin{array}{ccc} \alpha_{i1} & \alpha_{i2} & \cdots \\ \vdots & \vdots & \ddots \\ \alpha_{n1} & \alpha_{n2} & \cdots \end{array} & \begin{array}{ccc} \alpha_{ij} & \cdots & \alpha_{ip}\\ \vdots & \ddots & \vdots \\ \alpha_{nj} & \cdots & \alpha_{np} \end{array} \end{array}\right]\times [\begin{array}{c} x_{1}\\ \begin{array}{c} x_{2}\\ \begin{array}{c} \vdots \\ x_{i} \end{array}\\ \vdots \end{array}\\ x_{p} \end{array}].$$ (2)

Accordingly,

$$\zeta_{i}=\alpha_{i1}x_{1}+\alpha_{i2}x_{2}+\cdots +\alpha_{ij}x_{j}+\cdots +\alpha_{ip}x_{p},$$ (3)

where the *α*_*ij*_ represents the weight value, reflecting the vector contribution value of *x*_*j*_ to *ζ*_*i*_.

The *α*_*ij*_ is normalized as follows:

$$\alpha_{i1}+\alpha_{i2}+\cdots +\alpha_{ij}+\cdots +\alpha_{ip}=1.$$ (4)

Hence the linear combined score of PCA (*SCORE*_*PCA*_) is defined as

$${SCORE}_{PCA}=\left(\mu_{1}\zeta_{1}+\mu_{2}\zeta_{2}+\cdots +\mu_{i}\zeta_{i}+\cdots +\mu_{k}\zeta_{k}\right)/\sum_{i=1}^{k}\mu_{i},$$ (5)

where the *μ*_*i*_ represents the contribution weight of *ζ*_*i*_ where *μ*_*1*_>*μ*_*2*_>…>*μ*_*i*_>…>*μ*_*k*_ the total *SCORE*_*PCA*_ and *K* represents the number of *ζ*_*s*_ selected to retain (*K* < *P*), where *K* is determined in two ways: 1) selecting the *ζ*_*i*_ in which the *μ*_*i*_ value is greater than 1. 2) selecting the highest number of *ζ*_*s*_ in which the sum total ∑*μ* contribution is greater than 85%.

### Application

Here Table 2 presents the data used as the supplier’s attribute *X*_*ij*_, indicating that the selection criterion score *i* based on the business manager *j* will shape an *n×p* matrix *X*, in which *n* represents the number of the selected criteria and *p* denotes the number of business managers.

Thus, the matrix *X* is entered into the PCA calculation (using SPSS), and a set of *ζ*_*s*_ is developed. The first five *ζ*_*s*_ will have 86.278% cumulative percentage which is higher than 85 percent of total variance. Hence the variance percentage of extraction sums of squared loadings of the first five *ζ*_*s*_ will be used in Eq (5) in order to develop the *SCORE*_*PCA*_ of individual selection criterion. At this point, the optimum supplier criteria are selected based on the highest number.

Based on the results of these 24 cases, *c*_*19*_ (certificate of GMP), *c*_*4*_ (product quality), *c*_*1*_ (product price), *c*_*18*_ (past record documentation) and *c*_*9*_ (CRM) have the highest *SCORE*_*PCA*_ among the other criteria and they are selected as the best criteria by the scores 6.390, 6.305, 6.147, 5.997 and 5.700, respectively.

Since GMP is the primary condition for selecting the supplier and all firms must have this certificate, we therefore, set aside this criterion for the rest of the survey.

Next section reviews Z-TOPSIS. This method, using the scores resulted from the PCA method, will be applied to obtain the importance value of each supplier with respect to each product.

### Z-TOPSIS method

A brief review of some principle definitions of fuzzy sets from Chen [31], Chen and Lee [32], and Sotoudeh-Anvari and Sadi-Nezhad [33] are given below.

## Definition 1: Fuzzy set

A fuzzy set *A* defined on a universe *X* can be represented as:

$$A=\{\left(x,\mu_{A}(x)\right)|x\in X\},$$ (6)

where the *μ*_*A*_(*x*): *X*→[0,1] is a membership function of *A*. The *μ*_*A*_(*x*) membership value can describe the belongingness degree of *x*∈*X* in *A*. In this study, the type-1 fuzzy number and Z-number have been represented in triangular fuzzy number form.

## Definition 2: Type-1 fuzzy number

A triangular fuzzy number $\tilde{A}=(a_{1},a_{2},a_{3})$ can be represented by the following membership function:

$$\mu_{\tilde{A}}(x)=\left\{ \begin{array}{l} \begin{array}{ll} 0, & x\in (-\infty ,a_{1})\\ \;\frac{x-a_{1}}{a_{2}-a_{1}}, & x\in [a_{1},a_{2}] \end{array}\\ \begin{array}{ll} \;\frac{a_{3}-x}{a_{3}-a_{2}}, & x\in [a_{2},a_{3}]\\ 0, & x\in (a_{3},+\infty ) \end{array} \end{array}\right.$$ (7)

## Definition 3: Z-number

The Z-number concept is associated with the reliability of the information. The Z-number is constituted from two components: $Z=(\tilde{A},\tilde{B})$, which *A* is the fuzzy number and $\tilde{B}$ represents the reliability of the fuzzy number, which is also given in fuzzy number [34].

Chen [31] proposed linguistic numbers in the form of Table 3 and Table 4. Additionally, Table 5 employs the Z-TOPSIS technique to address the reliability of DMs. The numbers in this table are proposed by the authors.

**Table 3:** Linguistic variable for importance weight of each criterion.

Linguistic variables	Triangular fuzzy number
Very Low (VL)	(0,0,0.1)
Low (L)	(0,0.1,0.3)
Medium Low (ML)	(0.1,0.3,0.5)
Medium (M)	(0.3,0.5,0.7)
Medium High (MH)	(0.5,0.7,0.9)
High (H)	(0.7,0.9,1)
Very High (VH)	(0.9,1,1)

**Table 4:** Linguistic variable for the ratings of all alternatives.

Linguistic variables	Triangular fuzzy number
Very Poor (VP)	(0,0,1)
Poor (P)	(0,1,3)
Medium Poor (MP)	(1,3,5)
Medium (M)	(3,5,7)
Medium Good (MG)	(5,7,9)
Good (G)	(7,9,10)
Very good (VG)	(9,10,10)

**Table 5:** Linguistic variables for the expert’s reliability.

Linguistic variables	Triangular fuzzy number
Strongly Unlikely (SU)	(0,0,0.1)
Unlikely (U)	(0,0.1,0.3)
Somewhat Unlikely (SWU)	(0.1,0.3,0.5)
Neutral (N) (0.3,0.5,0.7)	(0.3,0.5,0.7)
Somewhat Likely (SWL)	(0.5,0.7,0.9)
Likely (L)	(0.7,0.9,1)
Strongly Likely (SL)	(0.9,1,1)

In order to determine the alternatives' ranking order, the following algorithm is operated, whereby the Step 1 is adopted from Kang *et al*. [35]; however it must be noticed that for the expert’s reliability, it uses the linguistics variable presented in Table 5 for the component B in Z-number, followed by Steps 2–7 from Chen [31].

## Step 1: Using the information obtained from the Table 5, the component $\tilde{B}$ is derived and then the Z-Number is converted to Type-1 fuzzy number

Assume a Z-number, $Z=(\tilde{A},\tilde{B})$. Suppose that $\tilde{A}=\{\left(x,\mu_{\tilde{A}}\right)|x\in [0,1]\},\;\tilde{B}=\{\left(x,\mu_{\tilde{B}}\right)|x\in [0,1]\},\;\mu_{\tilde{A}}$ and $\mu_{\tilde{B}}$ are triangular membership functions. The second part (reliability) should be converted into crisp number through fuzzy expectation as presented in Eq (8).

$$\alpha =\frac{\int x\mu_{\tilde{B}}dx}{\int \mu_{\tilde{B}}dx},$$ (8)

where the *∫* represents an algebraic integration. Afterwards, the second part weight–the reliability–should be added to the first part. Eq (9) shows the weighted Z-number.

$${\tilde{Z}}^{\alpha }=\{\left(x,\mu_{{\tilde{A}}^{\alpha }}\right)|\mu_{{\tilde{A}}^{\alpha }}(x)=\alpha \mu_{{\tilde{A}}^{\alpha }}(x),x\in \left[0,1\right]\}$$ (9)

It can be type-1 fuzzy number as presented by the Eq (10).

$${\tilde{Z}}^{\prime }=\{\langle x,\mu_{{\tilde{Z}}^{\alpha }}\rangle |\mu_{{\tilde{Z}}^{\alpha }}\left(x\right)=\alpha \mu_{{\tilde{A}}^{\alpha }}(\frac{x}{\sqrt{\alpha }}),x\in \left[0,1]\right\}$$ (10)

As can be understood from Kang *et al*. [35], the ${\tilde{Z}}^{\prime }$ features are similar fuzzy expectation to ${\tilde{Z}}^{\alpha }$.

## Step 2: Construct decision matrix, $\tilde{D}$ and weight matrix, $\tilde{W}$

Suppose that a decision making group is having *K* number of people; here the criteria importance and the alternatives pertaining to the individual criterion must be calculated using the Eq (11).

$$\begin{array}{l} {\tilde{x}}_{ij}=\frac{1}{K}[{\tilde{x}}_{ij}^{1}\left(+\right){\tilde{x}}_{ij}^{2}\left(+\right)\cdots \left(+\right){\tilde{x}}_{ij}^{K}],\\ {\tilde{w}}_{j}=\frac{1}{K}\left[{\tilde{w}}_{j}^{1}\left(+\right){\tilde{w}}_{j}^{2}\left(+\right)\cdots \left(+\right){\tilde{w}}_{j}^{K}\right], \end{array}$$ (11)

where the ${\tilde{x}}_{ij}$ and ${\tilde{w}}_{j}$ represent the rating and the importance weight of *K*^*th*^ decision maker.

As can be seen from the Eq (12), the multi-criteria decision making problem can readily be explained in matrix format.

$$\begin{array}{l} \tilde{D}=\left[\begin{array}{cc} \begin{array}{cc} {\tilde{X}}_{11} & {\tilde{X}}_{12}\\ {\tilde{X}}_{21} & {\tilde{X}}_{22} \end{array} & \begin{array}{cc} \cdots & {\tilde{X}}_{1n}\\ \cdots & {\tilde{X}}_{2n} \end{array}\\ \begin{array}{cc} \vdots & \vdots \\ {\tilde{X}}_{m1} & {\tilde{X}}_{m2} \end{array} & \begin{array}{cc} \ddots & \vdots \\ \cdots & {\tilde{X}}_{mn} \end{array} \end{array}\right]\\ \tilde{w}=\left[\begin{array}{ccc} {\tilde{w}}_{1} & \begin{array}{cc} {\tilde{w}}_{2} & \cdots \end{array} & {\tilde{w}}_{n} \end{array}\right], \end{array}$$ (12)

where ${\tilde{x}}_{ij}$ for all *i*, *j* and ${\tilde{w}}_{j},\;j=1,2,\ldots ,n$ constitute the linguistic variables. The above linguistic variables are described using the fuzzy numbers, ${\tilde{x}}_{ij}=(a_{ij},b_{ij},c_{ij})$ and ${\tilde{w}}_{ij}=(w_{j1},w_{j2},w_{j3})$.

## Step 3: Construct normalized fuzzy decision matrix, $\tilde{R}$

To make different scales comparable, the linear scale transformation shall be used to create the normalized decision making matrix as represented in Eq (13).

Suppose that

$$\tilde{R}={\left[{\tilde{r}}_{ij}\right]}_{m\times n},$$ (13)

where *B* and *C* respectively are the set of benefit and cost criteria, and

$$\begin{array}{l} {\tilde{r}}_{ij}=\left(\frac{a_{ij}}{c_{j}^{*}},\frac{b_{ij}}{c_{j}^{*}},\frac{c_{ij}}{c_{j}^{*}}\right),j\in B;\\ {\tilde{r}}_{ij}=(\frac{a_{j}^{-}}{c_{ij}},\frac{a_{j}^{-}}{b_{ij}},\frac{a_{j}^{-}}{a_{ij}}),j\in C;\\ c_{j}^{*}=\max_{i}\;c_{ij}\;\mathrm{if}\;j\in B;\\ a_{j}^{-}={min}_{i}\;a_{ij}\;\mathrm{if}\;j\in C. \end{array}$$ (14)

The above mentioned technique is intended to retain the feature that the ranges of normalized fuzzy numbers are in the interval [0,1].

## Step 4: Construct weighted normalized fuzzy decision matrix, $\tilde{V}$

Given the different importance of each individual criterion, constructing the weighted normalized fuzzy decision matrix would be possible using the Eq (15).

$$\tilde{V}={\left[{\tilde{v}}_{ij}\right]}_{m\times n}\;i=1,2,\ldots ,m\;and\;j=1,2,\ldots ,n,$$ (15)

where ${\tilde{v}}_{ij}={\tilde{r}}_{ij}(.){\tilde{w}}_{j}$.

## Step 5: Find fuzzy positive-ideal solution, A^*^ and fuzzy negative-ideal solution, *A*^*-*^

Next, we normalize all ${\tilde{v}}_{ij}$ in terms of some new triangular fuzzy numbers. Afterwards, the fuzzy positive ideal solution and fuzzy negative ideal solution are defined according to the Eq (16).

$$\begin{array}{l} A^{*}=({\tilde{\upsilon }}_{1}^{*},{\tilde{\upsilon }}_{2}^{*},\ldots ,{\tilde{\upsilon }}_{n}^{*}),\\ A^{-}=\left({\tilde{\upsilon }}_{1}^{-},{\tilde{\upsilon }}_{2}^{-},\ldots ,{\tilde{\upsilon }}_{n}^{-}\right), \end{array}$$ (16)

where ${\tilde{v}}_{j}^{*}=(1,1,1)$ and, ${\tilde{v}}_{j}^{-}=(0,0,0)$ for *j* = 1,2,…,*n*.

## Step 6: Find the distance of each alternative from A^*^ and *A*^*-*^

Now it would be possible to calculate the distance of each alternative from A^*^ and *A*^*-*^ in accordance with the Eq (17).

$$\begin{array}{l} d_{i}^{*}=\sum_{j=1}^{n}d\left({\tilde{\upsilon }}_{ij},{\tilde{\upsilon }}_{j}^{*}\right),\;i=1,2,\ldots ,m,\\ d_{i}^{-}=\sum_{j=1}^{n}d\left({\tilde{\upsilon }}_{ij},{\tilde{\upsilon }}_{j}^{-}\right),\;i=1,2,\ldots ,m, \end{array}$$ (17)

where *d*(.,.) is the distance measurement between two fuzzy numbers.

## Step 7: Find closeness coefficient, *CC*_*i*_

For the purpose of determining the ranking order of all alternatives, a closeness coefficient shall be defined when the $d_{i}^{*}$ and $d_{i}^{-}$ of each alternative *A*_*i*_ for *i* = 1,2,…,*m* have been calculated. To calculate the closeness coefficient of each individual alternative, the Eq (18) is used.

$${CC}_{i}=\frac{d_{i}^{-}}{d_{i}^{*}+d_{i}^{-}},\;i=1,2,\ldots ,m$$ (18)

It is obvious that by the approaching of *CC*_*i*_ to 1, an alternative *A*_*i*_ gets closer to A^*^ and farther from *A*^*-*^. Hence, based on the closeness coefficient, the ranking order of all alternatives can be determined and from a set of feasible alternatives, the best one can be selected.

In the next section, a new model is presented to determine the suppliers and the amount of the products provided from the related suppliers.

### The proposed model

The proposed methodology consists of three steps. In the first step, using the PCA method, the number of supplier selection criteria is reduced. In the next step, using the scores resulted from the PCA method, the importance value of each supplier is obtained via Z-TOPSIS with respect to each item. Then, these values are used in the mixed integer linear programming (MILP) model [36] as explained below.

### Assumptions

1. In this study, the purchaser would be allowed to buy from several suppliers.
2. A multi-product environment is considered for this study and several products can be supplied for the customer by the suppliers.
3. The product amounts and the number of suppliers are known.
4. The buyer is allowed to buy for only one single period.
5. Demand has been considered as constant, without any change during the planning period.
6. A fixed budget for purchasing all the products has been considered.

### Indices

*i* and *j* represent the suppliers and the products, respectively.

### Parameters

*d*_*ij*_ The mean of defective items of product *i* purchased from supplier *j*

*SIV*_*ij*_ The Importance value of supplier *j* pertinent to item *i* resulting from the Z-TOPSIS method

*P*_*ij*_ Price of product *i* purchased from supplier *j*

*B*_*T*_ The total amount of budget available for procurement of various products.

*D*_*i*_ Demand for product *i*

*S*_*ij*_ Minimum capacity of product *i* purchased from supplier *j*

$S_{ij}^{\prime }$ Maximum capacity of product *i* purchased from supplier *j*

*R*_*ij*_ Minimum order of product *i* purchased from supplier *j*

$R_{ij}^{\prime }$ Maximum order of product *i* purchased from supplier *j*

*maxNS* Maximum number of possible suppliers to be selected

*minNS* Minimum number of possible suppliers to be selected

### Decision variables

*x*_*ij*_ The amount of product *i* purchased from supplier *j*

*y*_*ij*_ The binary variable, which is one in case the product *i* is purchased from the supplier *j* and will be zero, otherwise

### MILP model

$$\mathrm{Max}\;Z=\sum_{i=1}^{n}\sum_{j=1}^{n}{SIV}_{ij}x_{ij}$$ (19)

$$s.t.\;\sum_{i=1}^{n}\sum_{j=1}^{n}P_{ij}x_{ij}\leq B_{T}$$ (20)

$$\sum_{j=1}^{n}x_{ij}(1-d_{ij})\geq D_{i},\;i=1,\ldots ,m$$ (21)

$$S_{ij}y_{ij}\leq x_{ij}\leq S_{ij}^{\prime }y_{ij},\;i=1,\ldots ,m,j=1,\ldots ,n$$ (22)

$$R_{ij}y_{ij}\leq x_{ij}\leq R_{ij}^{\prime }y_{ij},\;i=1,\ldots ,m,j=1,\ldots ,n$$ (23)

$$minNS\leq \sum_{i=1}^{n}\sum_{j=1}^{n}y_{ij}\leq \max NS,$$ (24)

$$x_{ij}\geq 0,y_{ij}\in \{0,1\},\;i=1,\ldots ,m,j=1,\ldots ,n$$ (25)

The objective function is presented in Eq (19) which determines the highest importance value of the selected suppliers relative to each product item through maximizing the related expression.

In constraints (20). the sum of the required budget must be determined, Eq (21) is associated with demand, Eqs 22 and 23 determine whether an item, say the part *i* is ordered from supplier *j*, the ordered number of items must lie within the supplier capacity and the required demand, respectively. Finally, the selected number of suppliers must be restricted by a minimum and a maximum numbers given by Eq (24).

In the section, a numerical example is given to show applicability of the proposed model. Note that in the first step, the business managers of 34 pharmaceutical companies are asked to rate the 24 supplier selection criteria. Using the PCA method, these 24 supplier selection criteria are reduced to 4, which are: product quality, product price, past record documentation and CRM. Therefore, all the pharmaceutical companies can only consider these four criteria and the scores resulted from the PCA method are considered as input for the next steps for the implementation of Z-TOPSIS and MILP model. For the example of the proposed method, we consider one firm with two decision makers who used 4 criteria obtained from the previous method. In other words, all other pharmaceutical firms could use these 4 criteria for ranking purposes.

## A case study

In the present study, Microsoft Excel is used for the suppliers' ranking. The evaluation of ranking and the suppliers' weights processes are described below. In this paper, we have used the linguistic numbers given in Table 2 to evaluate the criteria importance; also we have used the information of Table 5 for criteria reliability measurement represented in Table 6 in the Z-number form.

**Table 6:** Importance of the criteria and the DM reliability.

	DM_1_		*DM*_2_	
	$\tilde{A}$	$\tilde{B}$	$\tilde{A}$	$\tilde{B}$
**Product quality**	VH	L	VH	SWL
**Product price**	H	SL	VH	SL
**Past record documentation**	H	SWL	M	L
**CRM**	MH	N	MH	SWL

Next, in order to evaluate the suppliers’ rating corresponding to each criterion, the DMs use the linguistic rating variable presented in Table 4 and make use of the data given in Table 5 for measuring the reliability of the supplier performance evaluation corresponding to each individual criterion as represented in Table 7 and Table 8.

**Table 7:** Rating of four suppliers by *DM*_*1*_ for all criteria.

	**Product quality**		**Product price**		**Past record documentation**		**CRM**	
	$\tilde{A}$	$\tilde{B}$	$\tilde{A}$	$\tilde{B}$	$\tilde{A}$	$\tilde{B}$	$\tilde{A}$	$\tilde{B}$
**Supplier 1**	MP	L	VG	L	MG	L	F	N
**Supplier 2**	MG	SL	G	SWL	MG	L	G	SWU
**Supplier 3**	F	SWL	G	L	G	L	MP	SWL
**Supplier 4**	G	SWL	G	SWL	F	SWL	MP	L

**Table 8:** Rating of four suppliers by *DM*_*2*_ for all criteria.

	**Product quality**		Product price		**Past record documentation**		**CRM**	
	$\tilde{A}$	$\tilde{B}$	$\tilde{A}$	$\tilde{B}$	$\tilde{A}$	$\tilde{B}$	$\tilde{A}$	$\tilde{B}$
**Supplier 1**	F	L	G	SL	G	SL	G	SWU
**Supplier 2**	MG	L	VG	SWL	F	L	G	N
**Supplier 3**	MG	N	MG	L	MG	L	F	SWL
**Supplier 4**	VG	SWL	G	SWL	MG	N	MP	L

Now for the purpose of supplier selection problem case study, the Z-TOPSIS Algorithm is applied. Table 9 below shows the final results:

**Table 9:** Suppliers ranking based on Z-TOPSIS.

Supplier	*CC*_*i*_	**Rank**
Supplier 1	0.474	3rd
Supplier 2	0.635	1st
Supplier 3	0.526	2nd
Supplier 4	0.354	4th

As we can observe from the results of Table 9, supplier 2 (*CC*_*2*_ = 0.635) is considered as the most important one followed by supplier 3 (*CC*_*3*_ = 0.526), supplier 1 (*CC*_*1*_ = 0.474) and supplier 4 (*CC*_*4*_ = 0.354).

These importance values are input to the proposed mathematical model for the coefficients of the objective function. The parameter values that are required for supplying one item are presented below.

$${SIV}_{11}=0.474;\;{SIV}_{12}=0.635;\;{SIV}_{13}=0.526;\;{SIV}_{14}=0.354;$$

$$P_{11}=2;\;P_{12}=2;\;P_{13}=4;\;P_{14}=5;\;B_{T}=2500;$$

$$d_{11}=0.020;\;d_{12}=0.016;\;d_{13}=0.050;\;d_{14}=0.010;\;D_{1}=100;$$

$$S_{11}=10;\;S_{12}=20;\;S_{13}=50;\;S_{14}=0;\;S_{11}^{\prime }=200;\;S_{12}^{\prime }=100;\;S_{13}^{\prime }=200;\;S_{14}^{\prime }=50;$$

$$R_{11}=0;\;R_{12}=0;\;R_{13}=0;\;R_{14}=0;\;R_{11}^{\prime }=100;\;R_{12}^{\prime }=50;\;R_{13}^{\prime }=200;\;R_{14}^{\prime }=200;$$

minNS=2;maxNS=3.

The mathematical model is coded in GAMS optimization program. The optimum solution and the amount of products that suppliers can provide are given below.

In order to code the mathematical model the GAMS optimization program has been used. The optimum solution and the product amounts that could be provided by the suppliers are shown below.

z=184.200;

$$x_{11}=100;\;x_{12}=50;\;x_{13}=200;\;x_{14}=0;$$

$$y_{11}=1;\;y_{12}=1;\;y_{13}=1;\;y_{14}=0.$$

## Sensitivity analysis

To investigate the effect of criteria weights on ranking of different suppliers, a sensitivity analysis is performed. Using varying degrees of criteria weights, resulted from the first step of Z-TOPSIS method, we have measured the changes in the outcome. Specifically, six cases were examined, but the ranking of the suppliers has remained unchanged. The details of the cases are presented in Table 10, and the sensitivity results for the suppliers 1 to 4 are shown in Table 11. The preliminary results have indicated that the proposed method could provide relatively robust results for supplier selection problem.

**Table 10:** Criteria weights according to different cases.

	**Current case**	**Case 1**	**Case 2**	**Case 3**	**Case 4**	**Case 5**	**Case 6**
**Product quality**	(0.803 0.893 0.893)	(0.767 0.856 0.856)	(0.730 0.820 0.820)	(0.694 0.783 0.783)	(0.657 0.747 0.747)	(0.621 0.710 0.710)	(0.584 0.674 0.674)
**Product price**	(0.800 0.950 1.000)	(0.764 0.914 0.964)	(0.727 0.877 0.927)	(0.691 0.841 0.891)	(0.654 0.804 0.854)	(0.618 0.768 0.818)	(0.581 0.731 0.781)
**Past record documentation**	(0.435 0.614 0.750)	(0.472 0.650 0.787)	(0.508 0.687 0.823)	(0.545 0.723 0.860)	(0.581 0.760 0.896)	(0.618 0.796 0.933)	(0.654 0.833 0.969)
**CRM**	(0.386 0.540 0.695)	(0.422 0.577 0.731)	(0.459 0.613 0.768)	(0.495 0.650 0.804)	(0.532 0.686 0.841)	(0.568 0.723 0.877)	(0.605 0.759 0.914)

**Table 11:** Sensitivity analysis results.

	**Current case**	**Case 1**	**Case 2**	**Case 3**	**Case 4**	**Case 5**	**Case 6**
**Supplier 1**	0.4741	0.477	0.479	0.482	0.484	0.486	0.488
**Supplier 2**	0.635	0.635	0.634	0.633	0.631	0.629	0.627
**Supplier 3**	0.526	0.526	0.525	0.525	0.524	0.523	0.522
**Supplier 4**	0.354	0.352	0.349	0.347	0.345	0.342	0.340

## Conclusions

Contrary to the broad spectrum of studies undertaken on the supplier selection, the supplier assessment and selection through the application of specific measures relevant to the pharmaceutical industry were not extensively investigated. To bridge the gap, the current study has introduced a new selection model for the under study company aiming at presenting the ideas on the set up procedure of a Z-TOPSIS based selection model particularly designed to resolve the supplier selection problem in pharmaceutical industry. However, for the other industries, the relevant characteristics and requirements must be primarily studied. The questionnaire used in this paper was strongly recommended for determining the tailor-made supplier selection criteria in pharmaceutical industry. Obviously, for other industries, the questionnaire must be updated based on different criteria.

The PCA method has been used in this study to cut the number of supplier selection criteria, coupled with MILP model based on the Z-TOPSIS method. The merits development was described below.

The PCA can effectively remove the multicollinearity existing among the criteria ranking and would help reducing the trade-offs and the errors frequency in the questionnaire. The subjective errors also could efficiently be reduced using the PCA, meaning that the weight allocated to each ζ would be generated automatically. This model avoids complex and subjective pair-wise comparison for determining the ranking weights, which would eventually result in mitigation of this type of subjective errors. Moreover, it reduces the dimensionality of the questionnaire data meanwhile retaining the significant amount of information.

TOPSIS method using Z-numbers is used in this paper through extending the fuzzy rule based approach in multi-criteria decision making analysis. The proposed method, meanwhile providing a more useful way of handling vagueness and imperfection of information in decision making process, represents the expert knowledge more precisely. This method is more efficient compared with the current non-rule based TOPSIS in relation with ranking process.

Moreover, due to the software availability (SPSS, Microsoft Excel and GAMS), the proposed model is considered a user-friendly tool applicable for supplier selection.

As a future study, it is possible to use recent advances of robust optimization for supplier selection and we leave it for interested researchers. Furthermore, it would be interesting if the future work discusses the relationship between the results of this paper and the ones in the quotient space of fuzzy numbers [37,38] or symmetric fuzzy numbers [39].

## Supporting information

S1 File English questionnaire. (PDF)

S2 File Farsi questionnaire. (PDF)
