# Supplementary material for: A supplier selection model in pharmaceutical supply chain using PCA, Z-TOPSIS and MILP: A case study
Source: PLoS One. 2018 Aug 15;13(8):e0201604. doi: 10.1371/journal.pone.0201604 (PMC6093669; doi:10.1371/journal.pone.0201604)
Supplement: S1 File — (PDF) [file pone.0201604.s001.pdf]

Supplier Selection in Pharmaceutical Supply Chain

In pharmaceutical companies, the main criteria are grouped under six categories, which are cost, quality, services, delivery, supplier profile and overall personnel capabilities. These primary criteria are decomposed into various sub-criteria that may affect the supplier selection.
Please rate these criteria from 0 (the least important) to 10 (the most important).

Cost

1. **Product price**
Mark only one oval.

	0	1	2	3	4	5	6	7	8	9	10	
the least important	⬭	⬭	⬭	⬭	⬭	⬭	⬭	⬭	⬭	⬭	⬭	the most important

2. **Payment terms**
Mark only one oval.

	0	1	2	3	4	5	6	7	8	9	10	
بی اهمیت	⬭	⬭	⬭	⬭	⬭	⬭	⬭	⬭	⬭	⬭	⬭	اهمیت خیلی زیاد

3. **Delivery cost**
Mark only one oval.

	0	1	2	3	4	5	6	7	8	9	10	
بی اهمیت	⬭	⬭	⬭	⬭	⬭	⬭	⬭	⬭	⬭	⬭	⬭	اهمیت خیلی زیاد

Quality

4. **Product quality**
Mark only one oval.

	0	1	2	3	4	5	6	7	8	9	10	
بی اهمیت	⬭	⬭	⬭	⬭	⬭	⬭	⬭	⬭	⬭	⬭	⬭	اهمیت خیلی زیاد

5.
The number of the defective items
Mark only one oval.

	0	1	2	3	4	5	6	7	8	9	10	
بی اهمیت	⬭	⬭	⬭	⬭	⬭	⬭	⬭	⬭	⬭	⬭	⬭	اهمیت خیلی زیاد

6.
Packaging and labeling
Mark only one oval.

	0	1	2	3	4	5	6	7	8	9	10	
بی اهمیت	⬭	⬭	⬭	⬭	⬭	⬭	⬭	⬭	⬭	⬭	⬭	اهمیت خیلی زیاد

7.
Quality management system certification
ISO 9001
Mark only one oval.

	0	1	2	3	4	5	6	7	8	9	10	
بی اهمیت	⬭	⬭	⬭	⬭	⬭	⬭	⬭	⬭	⬭	⬭	⬭	اهمیت خیلی زیاد

8.
Research, development and innovation
Mark only one oval.

	0	1	2	3	4	5	6	7	8	9	10	
بی اهمیت	⬭	⬭	⬭	⬭	⬭	⬭	⬭	⬭	⬭	⬭	⬭	اهمیت خیلی زیاد

Services

9.
Customer Relationship Management
CRM
Mark only one oval.

	0	1	2	3	4	5	6	7	8	9	10	
بی اهمیت	⬭	⬭	⬭	⬭	⬭	⬭	⬭	⬭	⬭	⬭	⬭	اهمیت خیلی زیاد

10.

After sales service/warranty

Mark only one oval.

	0	1	2	3	4	5	6	7	8	9	10	
بی اهمیت												اهمیت خیلی زیاد

Delivery

11.

Geographical location

Mark only one oval.

	0	1	2	3	4	5	6	7	8	9	10	
بی اهمیت												اهمیت خیلی زیاد

12.

On time delivery

Mark only one oval.

	0	1	2	3	4	5	6	7	8	9	10	
بی اهمیت												اهمیت خیلی زیاد

Supplier profile

13.

Financial status

Mark only one oval.

	0	1	2	3	4	5	6	7	8	9	10	
بی اهمیت												اهمیت خیلی زیاد

14.

Management and organization

Mark only one oval.

	0	1	2	3	4	5	6	7	8	9	10	
بی اهمیت												اهمیت خیلی زیاد

15. **Technical ability**

Mark only one oval.

	0	1	2	3	4	5	6	7	8	9	10	
بی اهمیت	◯	◯	◯	◯	◯	◯	◯	◯	◯	◯	◯	اهمیت خیلی زیاد

16. **Facilities**

Mark only one oval.

	0	1	2	3	4	5	6	7	8	9	10	
بی اهمیت	◯	◯	◯	◯	◯	◯	◯	◯	◯	◯	◯	اهمیت خیلی زیاد

17. **Capacity**

Mark only one oval.

	0	1	2	3	4	5	6	7	8	9	10	
بی اهمیت	◯	◯	◯	◯	◯	◯	◯	◯	◯	◯	◯	اهمیت خیلی زیاد

18. **Past record documentation**

Mark only one oval.

	0	1	2	3	4	5	6	7	8	9	10	
بی اهمیت	◯	◯	◯	◯	◯	◯	◯	◯	◯	◯	◯	اهمیت خیلی زیاد

19. **Certificate Good Manufacturing Practice**

GMP

Mark only one oval.

	0	1	2	3	4	5	6	7	8	9	10	
بی اهمیت	◯	◯	◯	◯	◯	◯	◯	◯	◯	◯	◯	اهمیت خیلی زیاد

20.
Environmental management system certification
ISO 14001
Mark only one oval.

	0	1	2	3	4	5	6	7	8	9	10	
بی اهمیت	◯	◯	◯	◯	◯	◯	◯	◯	◯	◯	◯	اهمیت خیلی زیاد

21.
Occupational health and safety management system certification
OHSAS 18001
Mark only one oval.

	0	1	2	3	4	5	6	7	8	9	10	
بی اهمیت	◯	◯	◯	◯	◯	◯	◯	◯	◯	◯	◯	اهمیت خیلی زیاد

22.
Risk assessment system
Mark only one oval.

	0	1	2	3	4	5	6	7	8	9	10	
بی اهمیت	◯	◯	◯	◯	◯	◯	◯	◯	◯	◯	◯	اهمیت خیلی زیاد

Overall personnel capabilities

23.
Labor overall skills
Mark only one oval.

	0	1	2	3	4	5	6	7	8	9	10	
بی اهمیت	◯	◯	◯	◯	◯	◯	◯	◯	◯	◯	◯	اهمیت خیلی زیاد

24.
Labor experience
Mark only one oval.

	0	1	2	3	4	5	6	7	8	9	10	
بی اهمیت	◯	◯	◯	◯	◯	◯	◯	◯	◯	◯	◯	اهمیت خیلی زیاد

Powered by
Google Forms
