# Supplementary material for: A supplier selection model in pharmaceutical supply chain using PCA, Z-TOPSIS and MILP: A case study
Source: PLoS One. 2018 Aug 15;13(8):e0201604. doi: 10.1371/journal.pone.0201604 (PMC6093669; doi:10.1371/journal.pone.0201604)
Supplement: S2 File — (PDF) [file pone.0201604.s002.pdf]

انتخاب تأمین کننده در زنجیره تأمین دارو

هدف از طراحی این پرسشنامه، بررسی میزان اهمیت معیارهایی است که شرکت های داروسازی جهت انتخاب شرکت های تأمین کننده در نظر می گیرند. مقتضی است درجه ی اهمیت هریک از معیارهای ذکرشده را از 0 تا 10 مشخص نمایید.

هزینه

1. **قیمت محصول**

Mark only one oval.

	0	1	2	3	4	5	6	7	8	9	10	
بی اهمیت	◯	◯	◯	◯	◯	◯	◯	◯	◯	◯	◯	[illegible]

2. **نحوه مطالبه و دریافت مبلغ قرارداد**

Mark only one oval.

	0	1	2	3	4	5	6	7	8	9	10	
بی اهمیت	◯	◯	◯	◯	◯	◯	◯	◯	◯	◯	◯	[illegible]

3. **هزینه حمل و نقل**

Mark only one oval.

	0	1	2	3	4	5	6	7	8	9	10	
بی اهمیت	◯	◯	◯	◯	◯	◯	◯	◯	◯	◯	◯	[illegible]

کیفیت

4. **کیفیت محصول**

Mark only one oval.

	10	9	8	7	6	5	4	3	2	1	0	
	⬭	⬭	⬭	⬭	⬭	⬭	⬭	⬭	⬭	⬭	⬭	بی اهمیت

5. **تعداد نقص (بازگشت محصول)**

Mark only one oval.

	10	9	8	7	6	5	4	3	2	1	0	
	⬭	⬭	⬭	⬭	⬭	⬭	⬭	⬭	⬭	⬭	⬭	بی اهمیت

6. **بسته بندی، نصب برچسب شناسایی کالا**

Mark only one oval.

	10	9	8	7	6	5	4	3	2	1	0	
	⬭	⬭	⬭	⬭	⬭	⬭	⬭	⬭	⬭	⬭	⬭	بی اهمیت

7. **گواهینامه سیستم مدیریت کیفیت**

ISO 9001

Mark only one oval.

	10	9	8	7	6	5	4	3	2	1	0	
	⬭	⬭	⬭	⬭	⬭	⬭	⬭	⬭	⬭	⬭	⬭	بی اهمیت

8. **تحقیق، توسعه و نوآوری**

Mark only one oval.

	10	9	8	7	6	5	4	3	2	1	0	
	⬭	⬭	⬭	⬭	⬭	⬭	⬭	⬭	⬭	⬭	⬭	بی اهمیت

خدمات

.9
مدیریت ارتباط با مشتری
CRM
.Mark only one oval

	0	1	2	3	4	5	6	7	8	9	10
بی اهمیت											

.10
خدمات پس از فروش
.Mark only one oval

	0	1	2	3	4	5	6	7	8	9	10
بی اهمیت											

تحویل

.11
فاصله مکانی تأمین کننده تا محل شرکت
.Mark only one oval

	0	1	2	3	4	5	6	7	8	9	10
بی اهمیت											

.12
تحویل به موقع
.Mark only one oval

	0	1	2	3	4	5	6	7	8	9	10
بی اهمیت											

ویژگی های تأمین کننده

13.
وضعیت مالی
.Mark only one oval

	0	1	2	3	4	5	6	7	8	9	10	
بی اهمیت	⬭	⬭	⬭	⬭	⬭	⬭	⬭	⬭	⬭	⬭	⬭	[illegible]

14.
مدیریت و سازماندهی
.Mark only one oval

	0	1	2	3	4	5	6	7	8	9	10	
بی اهمیت	⬭	⬭	⬭	⬭	⬭	⬭	⬭	⬭	⬭	⬭	⬭	[illegible]

15.
توانایی فنی
.Mark only one oval

	0	1	2	3	4	5	6	7	8	9	10	
بی اهمیت	⬭	⬭	⬭	⬭	⬭	⬭	⬭	⬭	⬭	⬭	⬭	[illegible]

16.
امکانات تولید
.Mark only one oval

	0	1	2	3	4	5	6	7	8	9	10	
بی اهمیت	⬭	⬭	⬭	⬭	⬭	⬭	⬭	⬭	⬭	⬭	⬭	[illegible]

17.
ظرفیت تولید
.Mark only one oval

	0	1	2	3	4	5	6	7	8	9	10	
بی اهمیت	⬭	⬭	⬭	⬭	⬭	⬭	⬭	⬭	⬭	⬭	⬭	[illegible]

18. **سیستم مستندات و مدارک پشتیبان**

Mark only one oval.

	0	1	2	3	4	5	6	7	8	9	10	
بی اهمیت												[illegible]

19. **گواهینامه روش خوب تولید**

GMP

Mark only one oval.

	0	1	2	3	4	5	6	7	8	9	10	
بی اهمیت												[illegible]

20. **گواهینامه سیستم مدیریت زیست محیطی**

ISO 14001

Mark only one oval.

	0	1	2	3	4	5	6	7	8	9	10	
بی اهمیت												[illegible]

21. **گواهینامه سیستم مدیریت ایمنی و بهداشت حرفه ای**

OHSAS 18001

Mark only one oval.

	0	1	2	3	4	5	6	7	8	9	10	
بی اهمیت												[illegible]

22.
سیستم ارزیابی ریسک
Mark only one oval.

	0	1	2	3	4	5	6	7	8	9	10	
بی اهمیت	⬭	⬭	⬭	⬭	⬭	⬭	⬭	⬭	⬭	⬭	⬭	[illegible]

منابع انسانی

23.
مهارت پرسنل
Mark only one oval.

	0	1	2	3	4	5	6	7	8	9	10	
بی اهمیت	⬭	⬭	⬭	⬭	⬭	⬭	⬭	⬭	⬭	⬭	⬭	[illegible]

24.
تجربه پرسنل
Mark only one oval.

	0	1	2	3	4	5	6	7	8	9	10	
بی اهمیت	⬭	⬭	⬭	⬭	⬭	⬭	⬭	⬭	⬭	⬭	⬭	[illegible]

نظرات و پیشنهادات

25.
با تشکر از زمانی که صرف پاسخگویی به سؤالات این پرسشنامه کردید. در صورتیکه در حین پاسخ با مشکل یا ابهامی مواجه شدید یا چنانچه اضافه نمودن معیاری ضرورت داشت، لطفاً بیان فرمایید.

Powered by

Google Forms
